# The Roles of SPOP in DNA Damage Response and DNA Replication

**DOI:** 10.3390/ijms21197293

**Published:** 2020-10-02

**Authors:** Masashi Maekawa, Shigeki Higashiyama

**Affiliations:** 1 Division of Cell Growth and Tumor Regulation, Proteo-Science Center, Ehime University, Toon 791-0295, Japan; shigeki@m.ehime-u.ac.jp; 2 Department of Biochemistry and Molecular Genetics, Ehime University Graduate School of Medicine, Toon 791-0295, Japan

**Keywords:** cullin-3, SPOP, DNA damage response, DNA repair, DNA replication, cancer, genome instability, topoisomerase

## Abstract

Speckle-type BTB/POZ protein (SPOP) is a substrate recognition receptor of the cullin-3 (CUL3)/RING type ubiquitin E3 complex. To date, approximately 30 proteins have been identified as ubiquitinated substrates of the CUL3/SPOP complex. Pathologically, missense mutations in the substrate-binding domain of SPOP have been found in prostate and endometrial cancers. Prostate and endometrial cancer-associated SPOP mutations lose and increase substrate-binding ability, respectively. Expression of these SPOP mutants, thus, causes aberrant turnovers of the substrate proteins, leading to tumor formation. Although the molecular properties of SPOP and its cancer-associated mutants have been intensively elucidated, their cellular functions remain unclear. Recently, a number of studies have uncovered the critical role of SPOP and its mutants in DNA damage response and DNA replication. In this review article, we summarize the physiological functions of SPOP as a “gatekeeper” of genome stability.

## 1. Introduction

Ubiquitination is a post-translational modification that results in the addition of ubiquitin molecules to a protein in eukaryotes. The glycine residue at the C-terminus of ubiquitin (a small, 76-amino-acid residue protein) is conjugated to a lysine residue of substrate proteins through the formation of an isopeptide bond [1]. Ubiquitin can bond to a substrate protein at varying locations, with each resulting modification forming a distinct conformation or code. Ubiquitin contains seven lysine residues (K6, K11, K27, K29, K33, K48, and K63) in its amino acid sequence, allowing for ubiquitination by other ubiquitin molecules, thereby forming a polyubiquitin chain [2]. When ubiquitin attaches to the N-terminus methionine of another ubiquitin, a linear ubiquitin chain forms [3]. These ubiquitin codes determine the physiological functions of each polyubiquitin chain (e.g., K48-polyubiquitination in proteasomal degradation, M1-polyubiquitination in NF-κB signaling, K11-polyubiquitination in signal transduction, K63-polyubiquitination in the DNA damage response, K33-polyubiquitination in membrane trafficking) [4,5,6,7,8].

Three different enzymes, E1 (ubiquitin-activating enzyme), E2 (ubiquitin-conjugating enzyme), and E3 (ubiquitin-ligating enzyme) are responsible for protein ubiquitination [9]. Mechanistically, the C-terminal glycine residues of ubiquitin form a high-energy thioester bond with a cysteine residue of E1 in an ATP-dependent manner, and then the activated ubiquitin is transferred to a cysteine residue of E2. The substrates E2-ubiquitin and E3 form a complex, and E3 catalyzes the transfer of ubiquitin from E2 to the substrates. The human genome encodes more than 600 E3 enzymes, and the research on ubiquitination shows that dysfunction of the ubiquitination process causes severe diseases, such as cancers, neurodegenerative diseases, muscle wasting, inflammatory disease, autoinflammation, and metabolic syndromes [10].

Ubiquitin ligases mainly fall into one of two classes: E3 ligase with HECT domain and E3 ligase with RING finger domain. Cullin-3 (CUL3) is a scaffold protein of the cullin (CUL)/RING-type E3 complex. Humans possess eight CUL proteins, CUL1, CUL2, CUL3, CUL4A, CUL4B, CUL5, CUL7, and CUL9 [11]. The CUL3/RING ubiquitin E3 complex consists of CUL3, substrate recognizing receptors (Bric-à-brac/Tramtrack/Broad complex domain-containing proteins (BTBPs), E2, and E3s (RBX1, ARIH1, DCNLs) [12,13]. BTBPs recognize their substrate proteins and interact with the N-terminus of CUL3, leading to their ubiquitination [14]. Modification of a ubiquitin-like protein called Nedd8 at lysine 712 of CUL3 is required for CUL3/BTBP-mediated ubiquitination [15]. The human genome contains 183 annotated BTBPs [16], which facilitate the ubiquitination of various substrates, making CUL3 necessary for fundamental cellular events and physiological development [17,18,19,20].

Speckle-type BTB/POZ protein (SPOP) is one of the BTBPs of the CUL3/RING type E3 complex. Approximately 10–15% of prostate cancer patients possess point mutations in the substrate-binding domain of SPOP [21]. SPOP mutants associated with prostate cancer fail to interact with and ubiquitinate their substrates, leading to the accumulation of oncogenic substrate proteins such as androgen receptor (AR), BRD2, and BRD4 [22,23,24,25]. In contrast, endometrial cancer-associated SPOP mutations increase the ability of substrate binding, leading to enhanced polyubiquitination of the substrate proteins, followed by their degradation [26,27]. The pathophysiological functions of the endometrial cancer-associated SPOP mutants are still controversial. Dysregulation of SPOP has also been reported in other cancers. Sequencing studies indicated that SPOP has variants in ovarian, liver and thyroid cancers [28,29,30,31,32]. The gene amplification of wild-type SPOP was detected in approximately 5% of breast cancer patients [33]. In the cytosol of clear-cell renal cell carcinoma (ccRCC), SPOP was overexpressed and mislocalized [34]. Together, the roles of SPOP in tumorigenesis is context-dependent. For example, SPOP functions as a tumor suppressor in prostate cancers, and may act as an oncogene in breast cancers and ccRCC.

The molecular properties of SPOP have been characterized and the substrates (~30 proteins) ubiquitinated by the CUL3/SPOP E3 complex have been identified [22]. However, the cellular functions of SPOP remain unclear. Recently, studies, including ours, have shown that SPOP plays crucial roles in both the replication and damage response of DNA [35,36,37,38,39,40]. In this review article, we specifically focus on the physiological significance of SPOP in homeostasis of the DNA integrity. Dysregulation of DNA damage response and DNA replication often generates genome instability, we thus suggest that SPOP prevents the generation of genome instability.

## 2. Molecular Properties of SPOP

**Figure 1:**
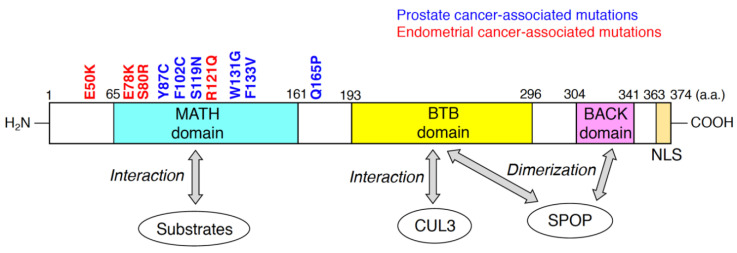
Domain structure of human speckle-type Bric-à-brac/Tramtrack/Broad complex (BTB)/POZ protein (SPOP) predicted by the Pfam database (https://pfam.xfam.org). Substrates of the cullin-3 (CUL3)/SPOP E3 complex directly bind to meprin and TRAF-C homology (MATH) domain of SPOP. The BTB domain of SPOP is responsible for the interaction with CUL3 and SPOP dimerization. BTB and C-terminal Kelch (BACK) domain is also required for SPOP dimerization. Representative missense mutations detected in prostate cancers and endometrial cancers are indicated in blue and red, respectively. The numbers in the domain structure represent the order of amino acids. The nuclear localization sequence (NLS) is also shown.

SPOP was originally discovered as a nuclear speckle-localizing protein in 1997 [41]. Subsequent biochemical and molecular analysis showed that SPOP serves as a substrate recognition receptor of the CUL3/RING-type E3 complex [22,42]. The human SPOP protein, consisting of 374 amino acid residues, possesses three domains (Figure 1). The meprin and TRAF-C homology (MATH) domain; Bric-à-brac/Tramtrack/Broad complex (BTB) domain; and BTB and C-terminal Kelch (BACK) domains are located at the N-terminal, central, and C-terminal regions of SPOP, respectively (Figure 1). The MATH domain is responsible for interactions with ubiquitinated substrates, and CUL3 binds to the BTB domain of SPOP [43]. The anti-parallel β-sheets in the MATH domain form a cleft to which the SPOP-binding (SB) motif binds [43]. The SB motif is a degron that consists of five amino acid residues, nonpolar-polar-S-S/T-S/T [44]. Both BTB and BACK domains are essential for the formation of linear SPOP oligomers [45,46]. At saturated concentrations of SPOP and substrates above a certain threshold, oligomers containing SPOP and substrates undergo liquid–liquid phase separation (LLPS) [47]. LLPS can enhance the efficient polyubiquitination of the substrates by the CUL3/SPOP complex [47]. In contrast, LLPS is disrupted by expression of prostate cancer-associated SPOP mutants [47], resulting in the decreased accessibility of substrates to the phase separation. Thus, the prostate cancer-associated SPOP mutants could exert dominant-negative effects. From a structural standpoint, SPOP is a unique protein because both the MATH and BTB domains can only be found in SPOP and its homologue in the human proteome, SPOP-like (SPOPL) [46,48]. SPOP possesses the nuclear localization sequence (NLS) at its C-terminus (Figure 1), which enables SPOP to localize at nuclear speckles. SPOP lacking its NLS localized at the puncta in cytosol [49]. Nuclear localization of SPOP would be necessary for its interactions with nuclear-localizing substrates.

A paradigm shift in the understanding of SPOP occurred when point mutations were identified in the MATH domain of patients with prostate cancer [21]. This discovery shed light on the pathophysiological significance of SPOP. The exosome sequences revealed that SPOP is heterogeneously mutated in approximately 10–15% of patients with prostate cancer [21,50]. Interestingly, these recurrent missense mutations were clustered in the substrate-interacting cleft of the MATH domain (Figure 1). Biochemically, prostate cancer-associated SPOP mutants (e.g., Y87C, and F133V) lose the ability to bind to substrates [22]. Since these mutants can interact with CUL3 as well as wild-type SPOP, the prostate cancer-associated SPOP mutants serve as dominant-negative mutants [51]. Thus, the heterogeneous expression of the prostate cancer-associated SPOP mutants reduced the polyubiquitination of substrates, followed by the inhibition of their proteasomal degradation. Because SPOP targets various oncogenic substrates (e.g., AR, DEK, TRIM24, NCOA3, BRD2, and BRD4), accumulation of these oncogenic proteins contributes to tumor formation in the prostate [52]. Another prostate cancer-associated mutation of SPOP, Q165P at the edge of the MATH domain, impairs dimerization of SPOP, resulting in the inhibition of substrate degradation [53].

Recent studies have shown that approximately 5% of patients with endometrial cancer also possess recurrent missense mutations in MATH domains [27] (Figure 1). In contrast to prostate cancer-associated SPOP mutants, the endometrial cancer-associated SPOP mutations are located outside the substrate-interacting cleft of the MATH domain [26]. Surprisingly, expression of SPOP mutants such as E50K and R121Q increased the polyubiquitination of some substrates (e.g., BRD2, BRD4, and NCOA3) [26]. These findings suggest that the endometrial cancer-associated SPOP mutants may have enhanced functional affinity to substrates and serve as gain-of-function mutants. The three-dimensional structures of SPOP mutants would differ between the prostate cancer-associated ones and the endometrial cancer-associated ones. Thus, co-crystallization analysis of various cancer-associated SPOP mutants and substrates revealed the structural mechanisms by which these SPOP mutants change their affinity to substrates.

## 3. Essential Functions of SPOP in the Homeostasis of DNA Integrity

Although the molecular characteristics of SPOP as well as a variety of ubiquitinated substrates of the SPOP/CUL3 E3 complex have been well elucidated, the pathophysiological cellular functions of both wild-type SPOP and cancer-associated SPOP mutants are still unclear. Recent discoveries have clearly indicated that SPOP is required for the resolution of DNA replication stress and the proper progression of DNA repair after DNA damage [35,36,37,38,39,40]. In this section, we summarize the recent research on the role of SPOP in the homeostasis of DNA integrity.

### 3.1. SPOP in DNA Damage Response

Cells are frequently exposed to both endogenous and exogenous DNA damage stress. Endogenous stressors include reactive oxygen species (ROS) and DNA replication stress (see Section 3.2). Exogenous stressors include chemicals, UV, and radiation. Both types of stressors result in the generation of DNA breaks such as single-strand breaks (SSBs) and/or DNA double-strand breaks (DSBs) [54,55]. Cells possess an evolutionally conserved response system to DNA damage stress called the DNA damage response (DDR), which is a sequential molecular cascade consisting of DDR sensors, mediators, transducers, and effectors. Generally, DDR sensor proteins such as the MRN complex and PARP1 accumulate on the DNA breaks [56]. A phosphorylated H2AX (γH2AX) is then accumulated on the DNA breaks as a consequence of the activation of DDR transducers (e.g., ATM, ATR, DNA-PK). The activation of the ATM/ChK2 pathway is a key process of the DDR for DSBs [57]. ATR functions in the process of responses for DNA replication stress (see Section 3.2). The final outputs of DDR (e.g., DNA repair, cell cycle arrest, apoptosis, and senescence) depend on DDR effectors. Among them, DNA repair is an essential processes of DDR termination. In particular, DSBs are restored through two major categories of DNA repairs: homology-directed repair (HDR) or non-homologous end joining (NHEJ) [58]. Error-free faithful HDR is active during the S or G2 phase [59]. HDR requires sequence homology between the donor and acceptor DNA. In contrast, in the process of error-prone NHEJ, nucleases resect damaged DNA at DSBs, and then polymerases synthesize new DNA chains, followed by ligation by ligases to repair the integrity of the DNA strands. It is well known that NHEJ induces gene rearrangement [60].

In 2014, Zhang et al. showed, for the first time, that SPOP is required for DNA repair in response to ionizing irradiation (IR) [40]. SPOP was partially relocalized to γH2AX-positive nuclear foci by treatment of cancerous cells (e.g., HeLa cells, U2OS cells, MDA-MB-231 cells) with IR or camptothecin, a topoisomerase 1 inhibitor [40]. The neutral gel comet assay revealed that SPOP knockdown delayed the repair of DSBs induced by irradiation in HeLa cells [40]. Although Zhang et al. suggested that SPOP interacts with ATM in response to IR-induced DNA damage [40], the molecular mechanisms by which SPOP terminates the repair of DSBs upon irradiation is unclear.

**Figure 2:**
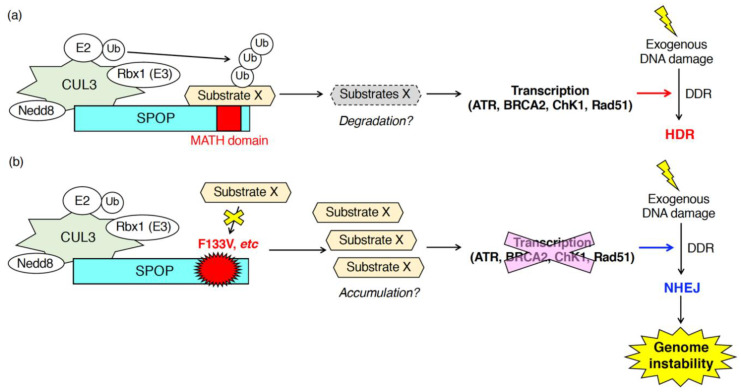
Patho-physiological roles of SPOP during the DNA damage response (DDR) in response to exogenous DNA damages. SPOP is essential for the mRNA expression of various DNA repair-related genes (ATR, BRCA2, ChK1, Rad51). (**a**) In cells expressing wild-type SPOP, ubiquitination of unidentified substrates promotes transcription of ATR, BRCA2, ChK1 and Rad51 leading to the proper DDR followed by error-free homology-directed repair (HDR) in response to exogenous DNA damages. (**b**) In cells expressing the F133V mutant of SPOP, inhibition of ubiquitination of substrates suppressed transcription of ATR, BRCA2, ChK1 and Rad51. Low expression of those genes promotes NHEJ after exposure to exogenous DNA damage leading to the generation of genome instability.

The clinical impact of prostate cancer-associated SPOP mutants, including F133V, on genome stability was investigated by using the whole genome sequence (WGS) database derived from prostate cancer patients [36]. The WGS analysis showed that gene rearrangements were detected more frequently in prostate cancers expressing SPOP mutants (*n* = 383) than in those expressing wild-type SPOP (*n* = 47). Since impaired DNA repair of DSBs frequently causes gene rearrangement [36], Boysen et al. hypothesized that SPOP functions in the DNA repair process after DSBs and expression of an F133V mutant alters the process (Figure 2). As expected, depletion of SPOP or expression of an F133V mutant suppressed HDR and instead enhanced NHEJ after introduction of DSBs by gamma irradiation or camptothecin treatment in prostate cancer cells and primary prostate epithelial cells [36]. In cells depleted of SPOP or expressing an F133V mutant, generation of Rad51-positive nuclear foci, a marker of HDR, was drastically suppressed and, by contrast, more 53BP1-positive nuclear foci, a marker of NHEJ, was generated after gamma irradiation [36]. It remains unclear whether 53BP1 is subjected to SPOP-dependent ubiquitination followed by its proteasomal degradation. Additional experiments such as immunoprecipitation and ubiquitination assay are required to examine the possibility. The promoted NHEJ and inhibited HDR are typical phenotypes of BRCA1-depleted cells, which are sensitive to PARP inhibitors [61]. Knockdown of SPOP or expression of prostate cancer-associated SPOP mutants sensitized prostate cancer cells to PARP inhibitor, olaparib, as was seen in BRCA1-depleted cells [36,61]. Similar changes in gene expression by BRCA1 depletion and by F133V mutant-expression were confirmed in a zebrafish model [36]. Gene rearrangement is a major cause of genome instability, a hallmark of cancer, including prostate cancer [62,63,64]. Together, these data strongly suggest that genome instability is a major cause of the development of prostate tumors that expresses an F133V mutant of SPOP. Essentially, the same phenotype in SPOP-manipulated prostate cancer cells was also reported by Bezawy et al. [37]. Expression of prostate cancer-associated SPOP mutants, Y87N, K129E, or F133V in DU145 cells and PC-3 cells sensitized to IR [37]. Importantly, prostate tumors expressing an F133V mutant were susceptible to IR in severe combined immunodeficiency (SCID) mice [37]. As reported previously [36], Rad51-positive nuclear foci were decreased in F133V-expressing DU145 cells upon IR [37].

Hjoth-Jensen et al. showed that SPOP knockdown reduced the generation of Rad51-positive cells upon camptothecin treatment and inhibited DNA replication in prostate cancer cells and human osteosarcoma U2OS cells [35]. Mechanistically, Hjoth-Jensen et al. suggested that SPOP is essential for the mRNA expression of DNA repair-related genes (e.g., ATR, BRCA2, ChK1, Rad51) [35]. SPOP knockdown in various prostate cancer cell lines (e.g., C4-2b cells, PC3 cells, LNCap cells, 22Rv1 cells) or U2OS cells reduced the mRNA and protein expression of ATR, BRCA2, ChK1, and Rad51 [35]. The reduced mRNA expression of those genes in SPOP-depleted cells was restored by exogenous expression of wild-type SPOP, but not by the expression of F133V mutant SPOP [35]. Hjoth-Jensen et al. performed proteomics to identify proteins that interact with wild-type SPOP but not with an F133V mutant [35]. Proteomics identified various wild-type specific interacting proteins that are involved in RNA splicing, nuclear export, and RNA pol II transcription [35]. However, the SPOP-dependent ubiquitination of SPOP-interacting proteins and their contributions to the regulation of mRNA expression of DNA repair-related genes are still unknown (Figure 2).

The critical roles of SPOP during the DDR upon exposure to exogenous DNA damage were also assessed in human lung adenocarcinoma cells [39]. As was seen in SPOP-depleted HeLa cells [40], SPOP knockdown significantly increased the formation of γH2AX-positive foci, and the mean comet tail moment in a neutral gel comet assay was higher than that in control H1299 cells after IR [39]. These data indicated that depletion of SPOP delayed the repair of IR-induced DSBs in human lung adenocarcinoma cells. SPOP knockdown increased the cell population in the G2/M phase and enhanced apoptosis of H1299 and A549 cells [39]. Dong et al. showed that SPOP depletion slightly increased the mRNA and protein expression of Rad51, a DSB-repair protein, in human lung adenocarcinoma cell lines [39]. However, it is still unclear whether this very small increase in Rad51 could contribute to the severe phenotypes (e.g., delay of DNA repair, cell cycle arrest, apoptosis, and suppression of cell growth) found in SPOP knockdown-lung cancer cells.

### 3.2. SPOP in DNA Replication

**Figure 3:**
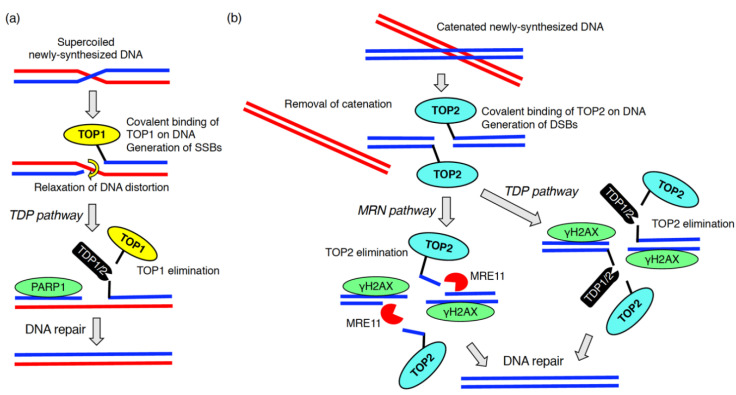
DNA–protein crosslink repair during DNA replication. To solve the topological issue of newly replicated DNA chains, topoisomerase 1 (TOP1) (**a**) or topoisomerase 2 (TOP2) (**b**) are covalently bound to the DNAs. After introduction of DNA breaks followed by the removal of DNA distortion, TOP1 and TOP2 are eliminated by enzymes, tyrosyl-DNA phosphodiesterase (TDP)1, TDP2 or meiotic recombination 11 (MRE11).

DNA replication is an essential process for all living organisms. During the replication, aberrant replication forks with uncoupled helicases and DNA polymerases are often generated. This complex phenomenon is defined as DNA replication stress [65]. The stalled replication forks lead to the formation of stretches of single stranded DNA, which is recognized by RPA followed by the activation of ATR [66]. DNA replication stress arises from a number of sources (e.g., lack of nucleotides, DNA lesions including DNA–protein adducts, DNA secondary structure, ribonucleotide incorporation) [65]. The generation of newly synthesized DNA chains in the S phase inevitably results in some endogenous DNA damage [67]. During this process, DNA replication stress caused by distortions in newly replicated DNA (e.g., supercoiled and catenated DNAs) is problematic [68]. To relieve these topological problems within DNA chains, topoisomerase 1 (TOP1) and topoisomerase 2 (TOP2) covalently bind to DNA leading to the formation of DNA–protein crosslinks (also called DNA–protein adducts) (Figure 3) [69,70]. TOP1 and TOP2 introduce SSBs and DSBs, respectively, resulting in the removal of supercoiled and catenated DNAs (Figure 3) [69]. After the resolution of DNA replication stress, TOP1 and TOP2 must be dissociated from the DNA–protein crosslink so proper DNA repair of the SSBs and DSBs can take place (Figure 3) [71]. This process is called DNA–protein crosslink repair [70]. During DNA–protein crosslink repair, the tyrosyl-DNA phosphodiesterase 1 (TDP1) or TDP2 eliminates TOP1 or TOP2 from the DNA–protein adducts. These enzymes cleave phosphotyrosyl bonds between the DNA and the tyrosine residue of TOP1 or TOP2, respectively (Figure 3) [69]. The endo/exonuclease, MRE11, which forms a complex with Rad50 and NBS1, also removes TOP2 from the DNA–protein adducts (Figure 3) [70]. MRE11 knockout increased the amount of the TOP2A-DNA cleavage complex [72].

**Figure 4:**
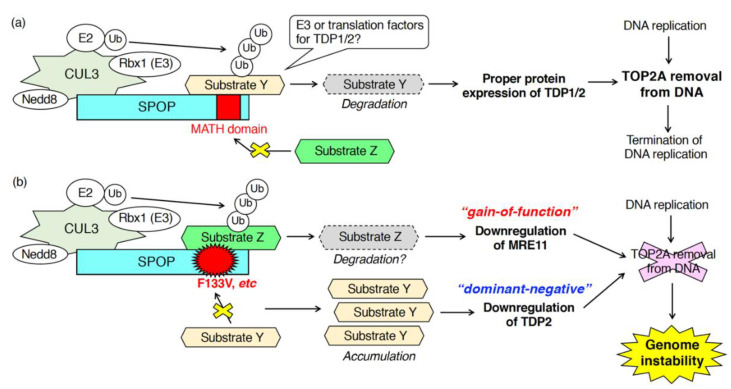
Patho-physiological roles of SPOP in DNA–protein crosslink repair during DNA replication. SPOP functions in the elimination of TOP2A from the TOP2A-DNA cleavage complex. (**a**) In cells expressing wild-type SPOP, the proper protein expression of TDP1 and TDP2 requires SPOP leading to the TOP2A removal from DNA. (**b**) In cells expressing F133V mutant of SPOP, the mutant serves as a dominant-negative and gain-of-function mutant in the downregulation of TDP2 and MRE11, respectively.

As described in Section 3.1, SPOP is essential for proper DDR upon exogenous DNA damage stress, such as radiation, UVs, hydroxyurea, camptothecin, irinotecan, etoposide [35,36,39,40]. In contrast, the function of SPOP in the DDR upon “endogenous” DNA damage stress in physiological conditions is unclear. To address this question, we examined DNA breaks in SPOP-depleted, normally growing, prostate cancer cells in the absence of any exogenous DNA damage stress [38]. Depletion of SPOP in AR-positive, prostate cancer C4-2 cells caused an accumulation γH2AX in the nuclei [38]. Upon SPOP depletion, activation of either ATM or ChK2 was not detected [38]. These data suggest that SPOP may control the activation of ATR when cells are not exposed to any exogenous DNA damage stress. We then examined the role of SPOP in the regulation of topoisomerase during DNA replication [38]. Both TOP1 and TOP2 are enzymatically active, and the protein expression of these proteins was not changed in SPOP-depleted C4-2 cells [38]. Notably, fluorescence intensity of topoisomerase 2A (TOP2A) but not TOP1 was significantly increased in SPOP-depleted C4-2 cells [38]. Biochemically, the amount of TOP2A-DNA cleavage complex was increased by SPOP knockdown [38]. These data suggest that SPOP is essential for the removal of TOP2A from the TOP2A-DNA cleavage complex during DNA replication. Mechanistically, SPOP depletion did not reduce the protein expression of meiotic recombination 11 (MRE11). However, it did reduce the protein expression of TDP1 and TDP2 without affecting their mRNA expression [38]. SPOP may interact with unidentified ubiquitin ligases for TDP1 and TDP2, leading to their degradation (Figure 4). Alternatively, SPOP may ubiquitinate unidentified substrates, which is essential for the proper translation of TDP1 and TDP2 (Figure 4). Depletion of TDP1 or TDP2 sensitizes cells to etoposide, a topoisomerase 2 inhibitor [73,74]. As expected, C4-2 cells depleted of SPOP or overexpressing wild-type SPOP were sensitive or resistant to the cytotoxicity of etoposide, respectively [38].

The accumulation of γH2AX by SPOP knockdown was observed in prostate cancer cell lines that were AR-positive (LNCaP cells, C4-2 cells), but not in those that were AR-negative (PC3 cells, DU145 cells) [38]. Treatment of SPOP-depleted C4-2 cells with the AR blocker enzalutamide suppressed the accumulation of γH2AX [38]. These data suggest that AR signaling activates TOP2A during DNA replication in prostate cancer cells, and SPOP-dependent removal of TOP2A from the TOP2A-DNA cleavage complex is necessary for the completion of DNA replication.

Expression of prostate cancer-associated SPOP mutants also caused the accumulation of DSBs in prostate cancer cells in the absence of exogenous DNA damage stresses [38]. Overexpression of prostate cancer-associated SPOP mutants (e.g., Y87C, and F133V) increased the amount of TOP2A, but not TOP1, in the nuclei as well as the drastic accumulation of γH2AX in the nuclei [38]. Transient overexpression of F133V in C4-2 cells decreased the protein expression of TDP2 and MRE11 [38]. Based on the observation that, in SPOP-depleted cells, protein expression of TDP2 was decreased, but MRE11 expression was unchanged, the F133V mutant appears to serve as a canonical dominant-negative and a novel gain-of-function mutant in the downregulation of TDP2 and MRE11, respectively (Figure 4) [38]. Mechanistically, to serve as a gain-of-function mutant, the F133V mutant may acquire the ability to bind to proteins with which the wild-type cannot (Figure 4). Further analysis is needed to elucidate the molecular properties of the F133V mutant, whose expression generates genome instability in prostate cancers.

Previous studies have shown that collaboration of TOP2A, TOP2B, and AR signaling efficiently induces DSBs, leading to gene rearrangements [75,76]. Gene rearrangements contribute to the tumor formation of prostate cancers by causing genome instability [62,63,64]. We suggest that SPOP maintains genomic stability through the proper removal of TOP2A from the TOP2A-DNA cleavage complex during DNA replication of normally growing cells. In cases where mutations are introduced in prostate-expressed SPOP (e.g., Y87C, F133V), these mutants lose the ability to remove TOP2A from DNA, resulting in genome instability. We suggest that other second hits (e.g., mutations in PTEN) in addition to the genome instability generated by the F133V mutants may cause the development of prostate cancers.

## 4. Conclusions and Perspectives

A line of studies has clearly indicated that the nuclear speckle-localizing protein, SPOP, is essential for various DNA repair processes in response to both exogenous and endogenous DNA damage stress. Expression of prostate cancer-associated mutants exhibits defects in these processes as well as depletion of wild-type SPOP. Delay and inhibition of the DNA repair process lead to the generation of genome instability, a hallmark of cancers. Together, SPOP serves as a “gatekeeper” to maintain genome stability. One big question that remains is: What are the ubiquitinated substrate proteins of SPOP that are responsible for the proper progress of DNA repair? Further proteome analysis is required to identify the bona fide substrates of the CUL3/SPOP E3 complex that function during the DNA repair process. A feature of SPOP in the regulation of the DNA repair process is that SPOP positively regulates the proper expression of various critical DNA repair factors at both the mRNA and protein levels (Figure 2 and Figure 4). SPOP is essential for the mRNA expression of ATR, BRCA2, ChK1, Rad51 [35] and for the protein expression of TDP1 and TDP2 [38]. It is likely that the CUL3/SPOP E3 complex ubiquitinates multiple substrates to properly progress the DNA repair. The spatiotemporal regulation of the interaction between SPOP and its substrates, followed by their ubiquitination, will be investigated in the future.

## Abbreviations

ATM	Ataxia telangiectasia mutated
ATP	Adenosine triphosphate
ATR	Ataxia telangiectasia and Rad3-related
AR	Androgen receptor
BACK	BTB and C-terminal Kelch
BTB	Bric-à-brac/Tramtrack/Broad complex
BTBPs	Bric-à-brac/Tramtrack/Broad complex (BTB) domain-containing proteins
BRCA1	Breast cancer susceptibility gene 1
BRD	Bromodomain containing
ccRCC	Clear-cell renal cell carcinoma
ChK1	Checkpoint kinase 1
ChK2	Checkpoint kinase 2
CUL	Cullin
DNA	Deoxyribonucleic acid
DNA-PK	DNA-dependent protein kinase
DDR	DNA damage response
DSB	Double-strand break
HDR	Homology-directed repair
IR	Ionizing irradiation
LLPS	Liquid-liquid phase separation
MATH	Meprin and TRAF-C homology
MRE11	Meiotic recombination 11
MRN	The Mre11-Rad50-Nbs1
mRNA	Messenger ribonucleic acid
NCOA3	Nuclear receptor coactivator 3
NHEJ	Non-homologous end joining
NLS	Nuclear localization sequence
PARP1	Poly[ADP-ribose] polymerase 1
PTEN	Phosphatase and tensin homologue deleted on chromosome 10
RNA pol II	RNA polymerase II
ROS	Reactive oxygen species
RPA	Replication protein A
SB	SPOP-binding
SCID	Severe combined immunodeficiency
SPOP	Speckle type BTB/POZ protein
SSB	Single-strand break
TDP	Tyrosyl-DNA phosphodiesterase
TOP1	Topoisomerase 1
TOP2	Topoisomerase 2
TRIM24	Tripartite motif containing 24
Ub	Ubiquitin
UV	Ultraviolet
WGS	Whole genome sequence
